# Real world effectiveness of Hawthorn special extract WS 1442 in a retrospective cohort study from Germany

**DOI:** 10.1038/s41598-024-74325-4

**Published:** 2024-10-03

**Authors:** Christophe Wyss, Peter W. Gündling, Karel Kostev

**Affiliations:** 1 HerzKlinik Hirslanden, Zürich, Switzerland; 2 Praxis für Naturheilverfahren, Bad Camberg, Germany; 3 Epidemiology, IQVIA, Unterschweinstiege 2–14, 60549 Frankfurt, Germany

**Keywords:** Hawthorn special extract WS 1442, Magnesium/potassium, Cardiac arrhythmias, Cardiology, Health care

## Abstract

Hawthorn special extract WS 1442 has beneficial effects on the cardiovascular system. Experimental studies have shown an antiarrhythmic effect of the substance. In the present study, we investigated antiarrhythmic effects of WS 1442 compared with magnesium/potassium in a large collective of outpatients. Using the IQVIA Disease Analyzer (DA) database, we included 4550 patients with a prescription of WS 1442 and 4550 matched patients with Tromcardin prescriptions (all registered products under the trademark Tromcardin that are magnesium and potassium supplementing foods for special medical purposes) who were followed for 5 years after the index date. The incidence of various cardiac arrhythmias (atrial fibrillation and flutter (AFF), tachycardia, and other cardiac arrhythmias) was recorded. Cox regression models were used to evaluate the potential association between both drugs and arrhythmias. The cumulative incidence of atrial fibrillation and flutter was significantly lower among patients with a prescription of WS 1442 compared to patients with magnesium/potassium prescriptions (10.8% vs. 16.4%, *p* < 0.001). WS 1442 prescription was significantly associated with a lower incidence of atrial fibrillation and flutter compared to magnesium/potassium (HR 0.71; 95% CI 0.64–0.80; *p* < 0.001). The cumulative incidence of tachycardia was significantly lower in the WS 1442 group compared to the magnesium/potassium group (8.3% vs. 9.4%, *p* < 0.001), similarly, the cumulative incidence of other cardiac arrhythmias was significantly lower among patients with WS 1442 compared to patients with magnesium/potassium (10.2% vs. 14.8%, *p* < 0.001). This study showed that in a large collective of outpatients, intake of hawthorn special extract WS 1442 was associated with a significantly lower incidence of atrial fibrillation, tachycardia, and other cardiac arrhythmias compared to magnesium/potassium, indicating its potential in treating and preventing such conditions.

## Introduction

Cardiovascular disease (CVD) is the most common cause of death worldwide. For example, each year more than 60 million potential years of life are lost to CVD in Europe^1^. Cardiovascular diseases include hypertension, coronary heart disease (CHD), angina pectoris (AP), atrial fibrillation (AF), heart failure, and others^2^. There are various evidence-based prevention strategies and treatments for the management of CVD, which include smoking reduction, weight reduction, physical activity, secondary prevention medications, and much more^3^. Along with synthetic drugs like beta blockers, diuretics, calcium channel blockers, ACE inhibitors, angiotensin II receptor blockers, lipid lowering drugs, and recently sodium glucose transporter 2 (SGLT2) inhibitors, hawthorn (Crataegus) has been playing an important role in the prevention and treatment of various cardiovascular diseases such heart failure for a very long time^4,5^.

Plant preparations made from hawthorn have various protective effects on the cardiovascular system, which are attributed to flavonoids and oligomeric procyanidins. These include positive inotropic effects, vasorelaxation, and improvement of the blood supply to the heart^6^. Hawthorn extracts further displayed negative chronotropic effects in a cultured neonatal murine cardiomyocyte assay. The preparations improved rhythmicity of the treated cardiomyocytes. However, it was shown that the chronotropic mechanism of action is not due to beta-adrenergic receptor blockade^7^. The antiarrhythmic effect of Crataegus extract on digoxin-induced arrhythmias was investigated in anesthetized Wistar rats and an antiarrhythmic effect was documented by shorter durations of atrial and ventricular arrhythmias in the treatment group compared to controls^8^. For WS 1442, a dry extract from hawthorn leaves with flowers (4-6.6:1), extraction solvent: ethanol 45% (w/w), a prolongation of the action potential and the refractory period as well as pronounced anti-arrhythmic properties could be shown^9^. Nonclinical studies thus support the traditional medicinal use of hawthorn preparations for the treatment of mild forms of arrythmias.

The positive effects of WS 1442 on the cardiovascular system was shown in various clinical and preclinical studies^10,11^. It strengthens the contractility of the heart and shows vasodilatative and vasoprotective effects in vitro. As a result, the oxygen supply can be increased^12^. In vivo studies showed a dose-dependent reduction of ventricular fibrillation and tachycardia in rats treated with WS 1442^13^. Zorniak et al.^13^ revealed the dose- and time-dependent cardioprotective effect of WS 1442 in rats including mortality index reduction as well as decrease in the incidence and duration of severe ventricular arrhythmias. Furthermore, a prospective, non-interventional study was conducted with patients from general practices^14^. In this study, the author compared 588 patients with mild heart failure (HF) who received WS 1442 and 364 patients who received therapy without hawthorn (comparative cohort). After 2 years of follow-up, the prevalences of fatigue, stress dyspnoea, and palpitations were significantly lower in the Crataegus cohort than in the comparative cohort.

Clinically, the antiarrhythmic and palpitation reducing effects of WS 1442 were first observed in heart failure patients during clinical studies^5,15,16^. Studies based on real-world data showing the antiarrhythmic effect of WS 1442 as active substance in terms of clinical outcomes have not been published yet. Another non-synthetic therapy used for cardiovascular prevention is Tromcardin. Under the tradename Tromcardin several products are marketed that contain magnesium and potassium and are regulatory classified as either complementary foods for special medical purposes or food supplements. The combination of magnesium and potassium may improve the electrolyte balance of the heart and may have an anti-arrhythmic effect by stabilizing the cell membrane and have thus a positive influence on contractility^17^. Since magnesium/potassium is prescribed as treatment of arrhythmia, it is used as a reference.

The present study aims to get insights on the impact of WS 1442 prescriptions on the incidence of atrial fibrillation and flutter (AFF), tachycardia, and other cardiac arrhythmias in a real-world setting.

## Methods

### Data source

This retrospective cohort study was based on the IQVIA Disease Analyzer database (DA), which contains case-based information provided by office-based physicians (both general practitioner (GPs) and specialists) in Germany. Information is available on patient demographics, drug prescriptions, concomitant medication, comorbid conditions, sick leave, and referrals to hospitals. Information is provided by nearly 3,000 office-based physicians, representing approximately 3% of all German practices. The database appears to be suitable for pharmaco-epidemiological and pharmaco-economic studies^18^.

### Ethical statement

German law allows the use of anonymous de-identified electronic medical records for research purposes under certain conditions. According to this legislation, it is not necessary to obtain informed consent from patients or approval from a medical ethics committee for this type of observational study that contains no directly identifiable data. Therefore, no waiver of ethical approval can be obtained from an Institutional Review Board (IRB) or ethics committee. The company and the authors involved had no access to any identifying information at any moment during the analysis of the data.

### Study population

This study included patients in GP practices with a prescription of the phytopharmaceutical WS 1442 or magnesium/potassium from January 2000 to December 2020. Patients with a diagnosis of atrial fibrillation and flutter (AFF) (ICD-10 I48), tachycardia and abnormalities of heart beat (ICD-10: I47 including re-entry ventricular arrhythmia, supraventricular tachycardia, ventricular tachycardia, and unspecified paroxysmal tachycardia, R00 including unspecified tachycardia, unspecified bradycardia, palpitations, other and unspecified abnormalities of heart beat), other cardiac arrhythmias (ICD-10: I49 including ventricular fibrillation and flutter, atrial premature depolarization, junctional premature depolarization, ventricular premature depolarization, other and unspecified premature depolarization, Sick sinus syndrome, extrasystoles, long-QT-syndrome and unspecified cardiac arrhythmia) in full patient history prior to the index date were excluded.

### Study outcomes and statistical analyses

Study outcomes were temporal associations between WS 1442 prescriptions and incidence of AFF, tachycardia, and other cardiac arrhythmias in the time period 30 days until 5 years following the index date as compared to magnesium/potassium as a reference group. Each patient was retrospectively followed for up to five years after index date till the first outcome diagnosis documentation, last patient’s visit, or end of database (December 31st, 2020). Patients prescribed with WS 1442 were matched to patients with a prescription of magnesium/potassium by sex and age.

To estimate the association between WS 1442 prescriptions and incidence of the above defined outcomes, Cox regression analyses were performed. Results of the Cox regression analysis are displayed using Hazard Ratios (HR) with 95% confidence intervals. These models were adjusted for diagnoses of obesity (ICD-10: E66), diabetes (ICD-10: E10-E14), lipid metabolism disorders (ICD-10: E78), heart failure (ICD-10: I50), renal failure (ICD-10: N18, N19), hypertension (ICD-10: I10), ischemic heart diseases (ICD-10: I20-I25), ischemic stroke (ICD-10: I63, I64, G45), tobacco addiction (ICD-10: F17), alcohol related disorders (ICD-10: F10), and chronic obstructive pulmonary disease (COPD) (ICD-10: J44), as well as co-prescriptions of diuretics (ATC: C03), betablockers (ATC: C08), calcium channel blockers (ATC: C08), and agents acting on the renin-angiotensin system (ATC: C09). As a sensitivity analysis, patients with WS 1442 prescription were matched to patients with a prescription of magnesium/potassium by sex, age, co-morbidities, and co-prescriptions. The association between WS 1442 prescriptions and incidence of the above defined outcome was analyzed using univariable Cox-regression analysis based on fully matched cohorts.

Differences in the sample characteristics between two therapy cohorts were compared using the Wilcoxon signed-rank test for continuous age, the Stuart-Maxwell test for categorical age, and the McNemar test for sex and comorbidities. P-values of < 0.05 were considered statistically significant. Regression analyses were repeated for several age groups, men, and women.

## Results

### Baseline characteristics

Initially, 5,304 patients with WS 1442 prescription and 15,515 patients with magnesium/potassium prescriptions were included. After matching of two cohorts, this study included 4,550 patients with WS 1442 prescription and 4,550 matched patients with magnesium/potassium prescriptions. Due to the matching, both cohorts were not significantly different in terms of average age (70.4 years) and sex (70–71% were females). The prevalence of heart failure (22.1% vs. 12.9%) was much higher among patients who started to be treated with WS 1442 than among patients who were put on magnesium/potassium. Diabetes (18.3% vs. 15.7%), lipid metabolism disorders (32.0% vs. 28.7%), and hypertension (52.1% vs. 46.5%) were more frequent among patients in the magnesium/potassium group. Moreover, prevalence of cardiovascular therapies, especially betablockers and agents acting on the renin–angiotensin system were more frequent in the magnesium/potassium group (Table 1).

**Table 1:** Characteristics of study patients after 1:1 matching.

Variable	Patients with WS 1442 (*n* = 4550)	Patients with magnesium/potassium (*n* = 4550)	*P*-value*
Age (mean, standard deviation)	70.4 (14.7)	70.4 (14.7)	1.000
≤ 60	26.8	26.8	1.000
61–70	19.0	19.0	
71–80	31.6	31.6	
> 80	22.6	22.6	
Female	70.9	70.4	0.644
Male	29.1	29.6	
Private health insurance	29.7	29.3	0.692
Statutory health insurance	71.3	71.7	
Obesity	5.9	6.6	0.106
Diabetes	15.7	18.3	< 0.001
Lipid metabolism disorders	28.7	32.0	< 0.001
Hypertension	46.5	52.1	< 0.001
Ischemic heart diseases	21.0	22.6	0.055
Heart failure	22.1	12.9	< 0.001
Ischemic stroke	4.6	4.7	0.887
Renal failure	5.1	5.7	0.155
COPD	8.0	8.9	0.113
Tobacco addiction	1.4	1.8	0.076
Alcohol related disorders	0.4	0.6	0.215
Drugs prescribed during the study therapy			
Diuretics	25.9	28.5	0.004
Betablockers	29.1	41.1	< 0.001
Calcium channel blockers	15.1	19.7	< 0.001
Agents acting on the renin–angiotensin system	39.8	49.2	< 0.001

Data are percentages unless otherwise specified.

*P-values were obtained using McNemar tests for categorical variables with two categories, Stuart-Maxwell tests for categorical variables with more than two categories, and Wilcoxon signed-rank tests for continuous variables.

### Incidence of atrial fibrillation and flutter

**Fig. 1:**
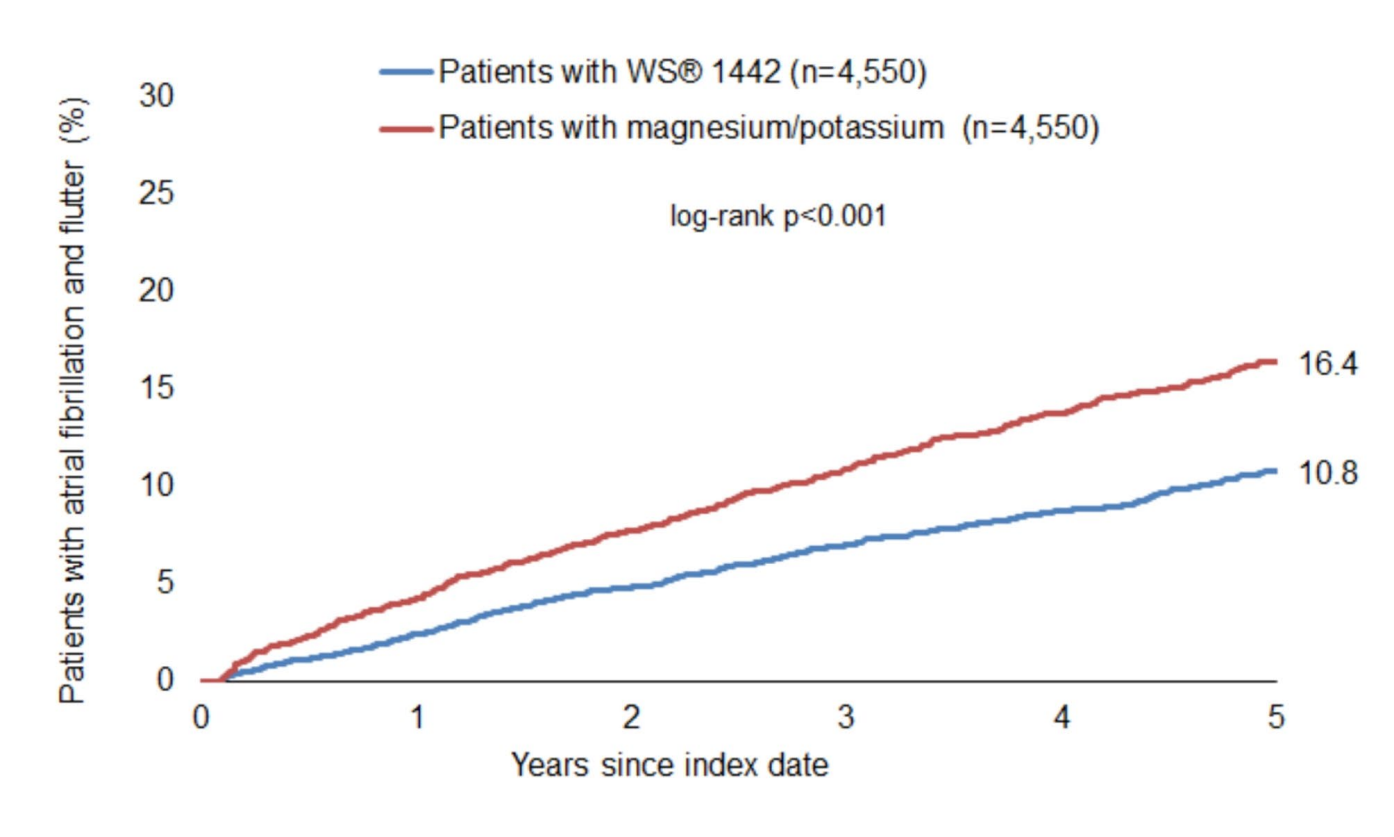
Cumulative incidence of atrial fibrillation and flutter in patients with WS 1442 and magnesium/potassium prescriptions.

The cumulative incidence of atrial fibrillation and flutter was significantly lower among patients with WS 1442 compared to those with magnesium/potassium (10.8% vs. 16.4%, *p* < 0.001) (Fig. 1), whereby each patient was retrospectively followed for up to five years after index date till the first outcome diagnosis documentation, last patient’s visit, or end of database. WS 1442 prescription was significantly associated with a lower incidence of atrial fibrillation and flutter compared to magnesium/potassium (HR: 0.71; 95% CI: 0.64–0.80; *p* < 0.001)—subgroup analyses showed consistent effects. These associations were confirmed in the sensitivity analysis (Table 2).

**Table 2:** Association between WS 1442 prescription as compared to magnesium/potassium prescription and incidence of atrial fibrillation and flutter.

Subgroup	Multivariable Cox regression adjusted for co-diagnoses and co-prescriptions* based on age- and sex-matched cohorts		Univariable Cox regression based on fully matched cohorts	
	Hazard ratio (95% CI)	*P*-value*	Hazard ratio (95% CI)	*P*-value*
Total	0.71 (0.64–0.80)	< 0.001	0.69 (0.61–0.77)	< 0.001
≤ 60	0.68 (0.50–0.92)	0.011	0.58 (0.37–0.91)	0.019
61–70	0.77 (0.59–1.01)	0.054	0.82 (0.64–1.06)	0.133
71–80	0.62 (0.52–0.75)	< 0.001	0.59 (0.49–0.71)	< 0.001
> 80	0.78 (0.63–0.97)	0.026	0.76 (0.62–0.94)	0.010
Female	0.65 (0.56–0.75)	< 0.001	0.59 (0.51–0.68)	< 0.001
Male	0.86 (0.71–1.05)	0.129	0.94 (0.78–1.15)	0.555

*Adjusted for obesity, diabetes, lipid metabolism disorders, hypertension, ischemic heart diseases, heart failure, ischemic stroke, renal failure, COPD, tobacco addiction, alcohol related disorders, and co-prescriptions of diuretics, betablockers, calcium channel blockers, agents acting on the renin–angiotensin system.

### Incidence of tachycardia

**Fig. 2:**
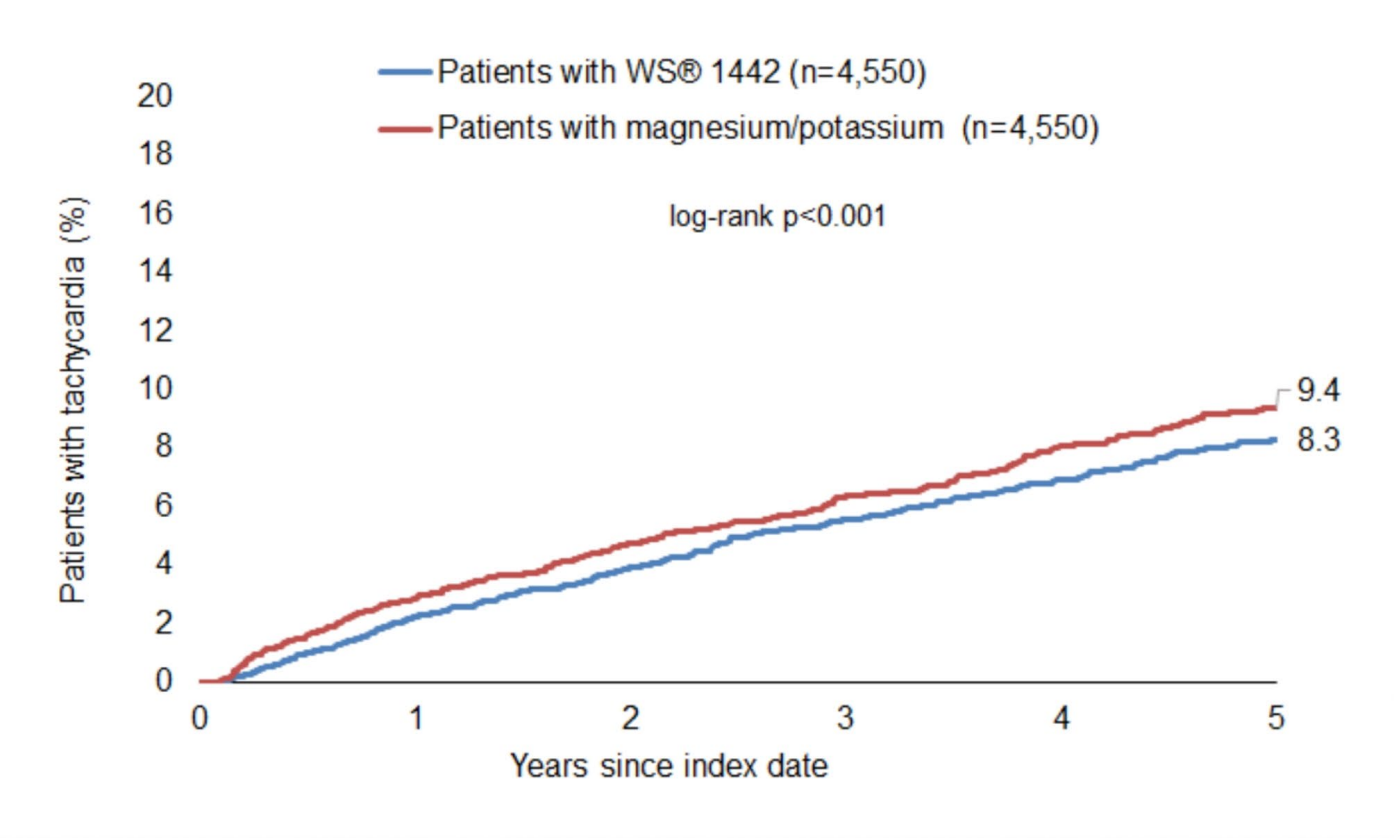
Cumulative incidence of tachycardia in patients with WS 1442 and magnesium/potassium prescriptions.

The cumulative incidence of tachycardia was significantly lower among patients with WS 1442 compared to patients with magnesium/potassium (8.3% vs. 9.4%, *p* < 0.001), however the absolute difference was relatively small (Fig. 2).

WS 1442 prescription was significantly associated with a lower incidence of tachycardia compared to magnesium/potassium (HR 0.83; 95% CI 0.73–0.96; *p* = 0.011). In subgroup analyses, the associations were consistent, but not always significant due to the reduced sample size. These associations were mostly confirmed in the sensitivity analysis (Table 3).

**Table 3:** Association between WS 1442 prescription as compared to magnesium/potassium prescription and incidence of tachycardia.

Subgroup	Multivariable Cox regression adjusted for co-diagnoses and co-prescriptions* based on age- and sex-matched cohorts		Univariable Cox regression based on fully matched cohorts	
	Hazard ratio (95% CI)	*P*-value*	Hazard ratio (95% CI)	*P*-value*
Total	0.83 (0.73–0.96)	0.011	0.91 (0.79–1.04)	0.168
≤ 60	0.73 (0.55–0.97)	0.031	0.76 (0.56–1.03)	0.075
61–70	0.87 (0.65–1.16)	0.343	0.90 (0.69–1.17)	0.438
71–80	0.82 (0.65–1.04)	0.098	0.98 (0.77–1.24)	0.840
> 80	0.90 (0.63–1.30)	0.586	0.97 (0.68–1.39)	0.884
Female	0.87 (0.74–1.03)	0.104	0.92 (0.78–1.08)	0.296
Male	0.72 (0.55–0.95)	0.021	0.87 (0.66–1.15)	0.322

*Adjusted for obesity, diabetes, lipid metabolism disorders, hypertension, ischemic heart diseases, heart failure, ischemic stroke, renal failure, COPD, tobacco addiction, alcohol related disorders, and co-prescriptions of diuretics, betablockers, calcium channel blockers, agents acting on the renin–angiotensin system.

### Incidence of other cardiac arrhythmias

**Fig. 3:**
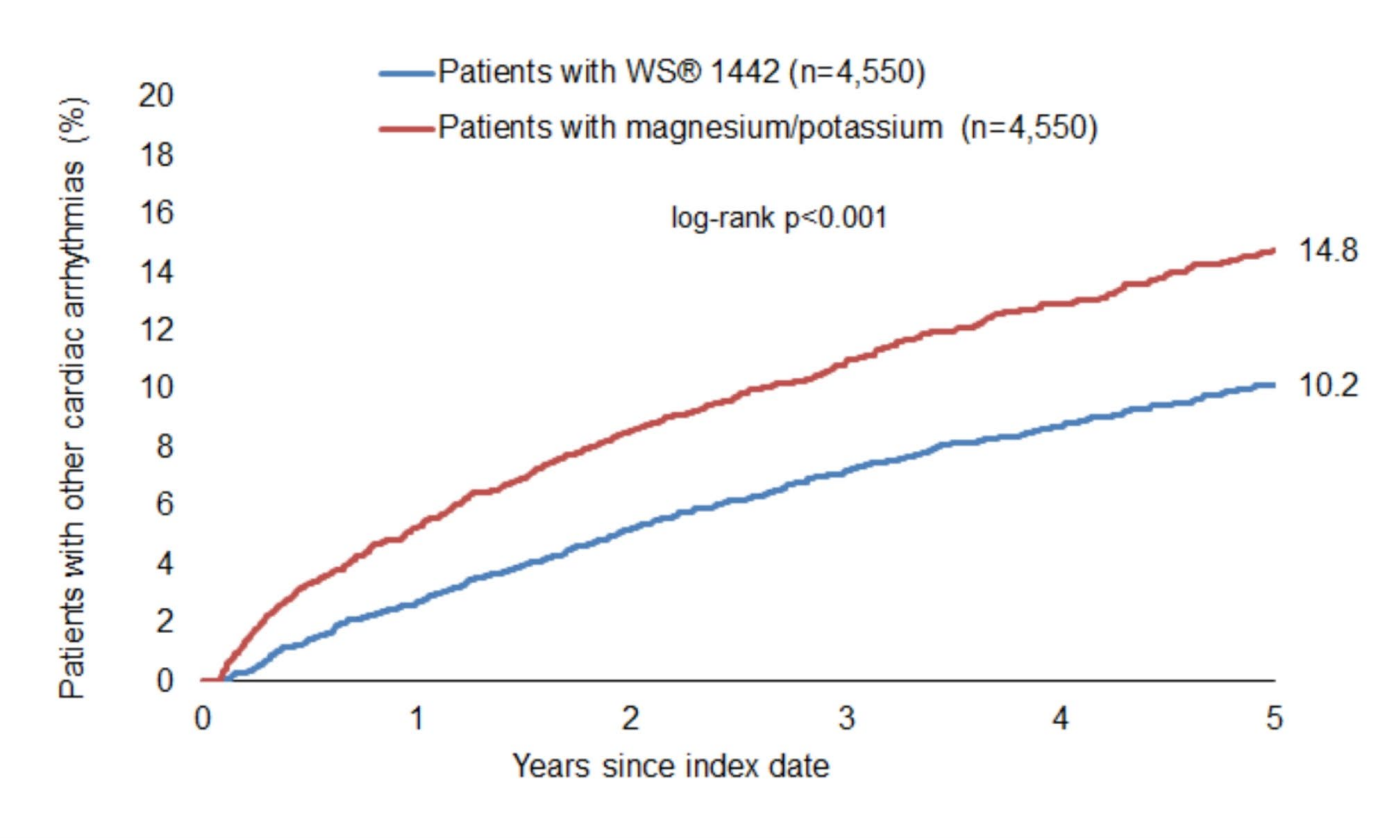
Cumulative incidence of other cardiac arrhythmias in patients with WS 1442 and magnesium/potassium prescriptions.

The cumulative incidence of other cardiac arrhythmias was significantly lower among patients with WS 1442 compared to patients with magnesium/potassium (10.2% vs. 14.8%, *p* < 0.001) (Fig. 3). WS 1442 prescription was significantly associated with a lower incidence of other cardiac arrhythmias compared to magnesium/potassium (HR: 0.68; 95% CI: 0.60–0.77; *p* < 0.001). This association was stronger among youngest (≤ 60) and oldest (> 80) age groups and also stronger among men compared to women. These associations were confirmed in the sensitivity analysis (Table 4).

**Table 4:** Association between WS 1442 prescription as compared to magnesium/potassium prescription and incidence of other cardiac arrhythmias.

Subgroup	Multivariable Cox regression adjusted for co-diagnoses and co-prescriptions* based on age- and sex-matched cohorts		Univariable Cox regression based on fully matched cohorts	
	Hazard ratio (95% CI)	*P*-value*	Hazard ratio (95% CI)	*P*-value*
Total	0.68 (0.60–0.77)	< 0.001	0.65 (0.58–0.74)	< 0.001
≤ 60	0.50 (0.38–0.65)	< 0.001	0.45 (0.33–0.53)	< 0.001
61–70	0.73 (0.57–0.93)	0.011	0.76 (0.61–0.96)	0.019
71–80	0.78 (0.63–0.97)	0.022	0.70 (0.57–0.85)	< 0.001
> 80	0.59 (0.44–0.80)	< 0.001	0.61 (0.46–0.80)	< 0.001
Female	0.72 (0.62–0.83)	< 0.001	0.67 (0.58–0.78)	< 0.001
Male	0.60 (0.49–0.75)	< 0.001	0.62 (0.50–0.76)	< 0.001

*Adjusted for obesity, diabetes, lipid metabolism disorders, hypertension, ischemic heart diseases, heart failure, ischemic stroke, renal failure, COPD, tobacco addiction, alcohol related disorders, and co-prescriptions of diuretics, betablockers, calcium channel blockers, agents acting on the renin–angiotensin system.

## Discussion

In this retrospective cohort study, we investigated antiarrhythmic effects of WS 1442 compared with magnesium/potassium in a large collective of outpatients. Using a matched-pairs design and based on more than 9,000 primary care patients, WS 1442 prescription was significantly associated with a lower incidence of three main heart rhythm disorders (atrial fibrillation and flutter, tachycardia, and other cardiac arrhythmias).

In previous clinical trials, WS 1442 has been compared to placebo. Mostly cardiovascular effects of WS 1442 among patients with chronic heart failure were investigated^16,19–21^. The largest clinical trial contained 2681 adults with NYHA class II/III heart failure (1338 treated with WS 1442). Using WS 1442 (900 mg/day) as an add-on to standard treatment, the authors demonstrated that WS 1442 could reduce the incidence of sudden cardiac death in patients with left ventricular ejection fraction between 25% and 35%^20^.

The use of WS 1442 and, ultimately, magnesium/potassium should not be seen as an alternative to guideline-based drug options for the prevention of cardiac arrhythmias. Rather, the former in particular seems to have an additional effect even in the presence of existing medication. It is known that WS 1442 is a therapy with few side effects and a favorable interaction profile. In the SPICE (Survival and Prognosis: Investigation of WS 1442 in congestive heart failure) study, administration of WS 1442 in combination with standard therapy for heart failure was associated with an excellent safety and tolerability profile, as the adverse events observed in the WS 1442 patients were of the same types and occurred at comparable rates as those in the placebo group^20^. It has been shown in vitro that the negative chronotropic effect of WS 1442 is unlikely to be promoted by a β-adrenergic receptor blockade as is the case with beta-blockers^22^.

The proportion of patients taking beta-blockers is relatively high in both comparison groups. Interestingly, beta-blockers, which have a clearly proven prophylactic effect for tachycardic cardiac arrhythmia of any kind^23^, were prescribed even less in the WS 1442 group than in the controls (29.1% vs. 41.1%). Furthermore, pre-existing heart failure was more present in the WS 1442 group (22.1% vs. 12.9%). Nevertheless, the incidence of various tachycardic cardiac arrhythmias is lower in this group, which could speak for the effect of WS 1442.

Different mechanisms have been mentioned as possible mediators of the cardioprotective effect of hawthorn: anti-inflammatory action, anti-oxidation, endothelial protection, anti-platelet action and prolongation of refractory period and action potential^13,24–27^. These are all mechanisms that are important in the pathogenesis of both heart failure and arrhythmias^28,29^. Interestingly, animal studies also show that WS 1442 may prevent ventricular arrhythmias, e.g., in ischemia-triggered arrhythmias. Oral administration of WS 1442 (10 or 100 mg x kg(-1) x day(-1)) for 7 days before ligation of the left coronary artery significantly decreased the area of myocardial infarction within the ischemic zone in rats^30^. WS 1442 treatment also attenuated the elevation of the ST-segment in the ECG, diminished the incidence of ventricular fibrillations (control: 67%; 10 mg x kg(-1): 64%; 100 mg x kg(-1): 27%) and reduced the mortality rate (control: 47%; 10 mg x kg(-1): 27%; 100 mg x kg(-1): 9%)^13^. Further studies would certainly be required to investigate the mechanism of action.

From a clinical perspective, there is a wide range of potential applications of WS 1442 in combination with the entire spectrum of therapeutic procedures, starting with basic measures such as exercise and a healthy nutrition, chemical-synthetic drug therapy, and interventional methods such as pulmonary vein isolation^23^. In clinical trials, an increase in quality of life was shown for WS 1442 in patients with heart failure NYHA II in combination with standard therapy and training compared to patients only receiving standard therapy and training^31^.

Retrospective primary care database analyses are generally limited by the validity and completeness of data. First, assessments are relied on ICD codes entered by GPs and no data from specialists or hospitals were available. Diagnosis codes lack deep granularity and do not allow for the differentiation of disease severity stages or outcomes. For example, outcome diagnoses were defined using ICD-10 3d level (i.e. I49) and no analyses of deeper levels (i.e. I49.1) were possible as the most frequent diagnoses by GPs are undefined (i.e. I49.9). Second, results of quality-of-life assessments, results of electrocardiogram (ECG), and data on socioeconomic status and lifestyle-related risk factors (smoking, alcohol, physical activity) are not available. Third, the prevalence of some cardiovascular disorders and therapies was more frequent among patients with magnesium/potassium. Although we used multivariable regression analyses to adjust for possible effects of co-diagnoses and co-therapies on the outcomes, the data may lack detailed diagnosis information that would allow for better adjustment. Fourth, to buy herbal medicines, which are OTC drugs, or dietary supplements, patients do not need a prescription from physicians. The database does not include data on the use of herbal medicines that patients buy without prescriptions. Fifth, since both preparations are OTC, data on adherence to therapy during the follow-up is not available. Finally, patients can only be observed in a single practice; when they receive a diagnosis or prescription by another physician, such prescriptions cannot be analyzed as data from different practices cannot be linked. However, the strengths of this study are the large sample size, the use of data collected in general practices representing a real-life context, and the inclusion of a wide range of physical conditions with adjustment for these in multivariable regression models.

## Conclusion

The findings of this retrospective cohort study showed that patients prescribed with WS 1442 exhibited a significantly lower incidence of atrial fibrillation and flutter, tachycardia, and other cardiac arrhythmias compared to those prescribed magnesium/potassium. These findings underscore the previously established use of WS 1442 for varying heart diseases as well as the importance of further exploration of WS 1442 as a promising adjunct in the treatment and prevention of cardiac arrhythmias.

## Data Availability

The data that support the findings of this study are not openly available due to reasons of privacy and are available from the corresponding author upon reasonable request.
